# Adapting Segment Anything Model 3 for concept-driven lesion segmentation in medical images: an experimental study

**DOI:** 10.1186/s42492-026-00234-0

**Published:** 2026-10-12

**Authors:** Guoping Xu, Jayaram K Udupa, Yubing Tong, Xin Long, Ying Zhang, Jie Deng, Weiguo Lu, You Zhang

**Affiliations:** 1 The Medical Artificial Intelligence and Automation (MAIA) Laboratory, Department of Radiation Oncology, University of Texas Southwestern Medical Center, Dallas, TX 75390 USA; 2 Medical Image Processing Group, Department of Radiology, University of Pennsylvania, Philadelphia, PA 19104 USA; 3 Division of Digestive and Liver Diseases, Department of Internal Medicine, University of Texas Southwestern Medical Center, Dallas, TX 75390 USA

**Keywords:** Vision-language foundation model, Prompt, Concept-driven segmentation, Medical image segmentation, Lesion

## Abstract

Accurate segmentation of lesions is fundamental for medical image analysis. However, most existing approaches are designed for specific anatomical sites and imaging modalities, limiting their applicability in heterogeneous clinical environments. Recent vision-language foundation models have enabled concept-driven segmentation of natural images, offering a promising path toward more flexible and generalizable medical image segmentation. However, to the best of the authors’ knowledge, concept-prompt-based lesion segmentation in medical images, particularly using the latest Segment Anything Model 3 (SAM3), has not been thoroughly examined. In this paper, the authors present a systematic investigation of the SAM3 framework for lesion segmentation. An extensive evaluation of SAM3 was first conducted using geometric bounding boxes and concept-based text and image exemplars as prompts for lesion segmentation across multiple cancer types and imaging modalities, including magnetic resonance imaging, computed tomography, ultrasound, dermoscopy, and endoscopic imaging, with particular emphasis on volumetric medical imaging datasets. To improve robustness and segmentation accuracy, additional knowledge into the input representation was further integrated, including predictions from adjacent slices, multiparametric imaging information, and prior annotations. In addition, the study compared different SAM3 fine-tuning strategies on the BraTS2023-MET dataset, including partial module fine-tuning, adapter-based optimization, and full-model fine-tuning, under different training objectives. Extensive experiments conducted on 13 datasets covering 11 lesion types demonstrate that SAM3 achieves strong cross-modality generalization and accurate instance-specific lesion delineation. These results highlight the potential of concept-driven foundation models for scalable and practical medical-image segmentation in real-world clinical settings. The source code and dataset splits are publicly available at https://github.com/apple1986/lesion-sam3.

**Supplementary Information:**

The online version contains supplementary material available at https://doi.org/10.1186/s42492-026-00234-0.

## Introduction

Medical image segmentation aims to accurately identify and delineate anatomical structures, tissues, or pathological lesions across diverse imaging modalities. It plays a critical role in numerous clinical applications, including diagnosis, treatment planning, disease monitoring, and quantitative assessment of pathological changes [1, 2]. In recent years, the rapid development of deep learning has led to substantial performance improvements in medical image segmentation, enabling an increasingly accurate and automated analysis of complex medical imaging data.

Generally, existing deep learning-based medical image segmentation methods have adopted different strategies to improve the segmentation accuracy and robustness: (1) Multiscale feature representation: this strategy is widely adopted to handle large variations in object size and is typically achieved through skip connections (e.g., U-Net [3] and Unet++ [4]), enlarged receptive fields via dilated convolutions or large kernels (e.g., the DeepLab series [5, 6] and SegNeXt [7]), or hierarchical feature pyramids constructed using pooling or strided convolutions (e.g., PSPNet [8] and UperNet [9]); (2) Local and global feature fusion: hybrid architectures that combine convolutional neural networks (CNNs) with transformer-based modules have been proposed to jointly capture fine-grained local details and long-range contextual dependencies, as exemplified by TransUNet [10], LeViT-Unet [11], and Swin-Unet [12]; (3) Prior-knowledge enhancement: prior-knowledge integration has been explored to further improve segmentation performance [13], including extending 2D slice-based segmentation to 3D volume-based frameworks to exploit spatial continuity and shape information (e.g., UNETR [14] and UNETR++ [15]), employing advanced data augmentation strategies [16], and introducing deep supervision by injecting label information at multiple stages of the decoder [17]; and (4) Other complementary approaches: additional methods have investigated novel loss functions [18, 19], uncertainty modeling [20], semi-supervised learning [21], and related techniques to further enhance robustness and generalization in challenging clinical scenarios [22, 23].

Although these approaches or techniques demonstrate impressive segmentation performance, they are often tailored to specific structures within a fixed anatomical site, such as the brain, thorax, or abdomen, and a single imaging modality, such as magnetic resonance imaging (MRI) or computed tomography (CT). Consequently, these models typically require retraining or extensive fine-tuning when applied to different anatomical sites or imaging modalities and often struggle to generalize effectively across the diverse and heterogeneous clinical environments encountered in real-world medical practice [24]. Driven by the remarkable success of large language models (LLMs), recent research has highlighted the effectiveness of scaling laws, wherein training transformer-based architectures with billions of parameters on massive datasets leads to substantial improvements in generalization and task adaptability [25, 26]. Inspired by these advances, large-scale vision foundation models have been actively investigated by leveraging unprecedented amounts of training data, increased model capacity, and novel training strategies to learn more discriminative visual representations [27, 28].

In the context of image segmentation, vision foundation models aim to provide a unified and transferable framework capable of adapting to diverse segmentation tasks with a high generalization ability and robustness in real-world scenarios. A representative example is the Segment Anything Model (SAM) introduced in 2023 [29]. Trained on a large-scale dataset comprising 11 million images and over 1 billion masks, SAM demonstrates remarkable generalization capability and strong segmentation performance on natural images under a prompt-based segmentation paradigm. Building on its architecture, subsequent studies have extended SAM to the medical imaging domain through large-scale training on medical image datasets, including MedSAM for 2D medical image segmentation [30] and SAM-Med3D for volumetric data [31]. In 2024, its successor SAM2 was proposed [32], which extended promptable segmentation from static images to videos. By incorporating streaming memory modules to leverage features and predictions from previous frames, SAM2 enables efficient and real-time multi-object video segmentation using simple geometric prompts such as points, bounding boxes, or scribble masks. Further studies, including DD-SAM2 [33] and TSMS-SAM2 [34], fine-tuned SAM2 on medical video datasets and demonstrated its strong performance in various clinical scenarios.

**Fig. 1:**
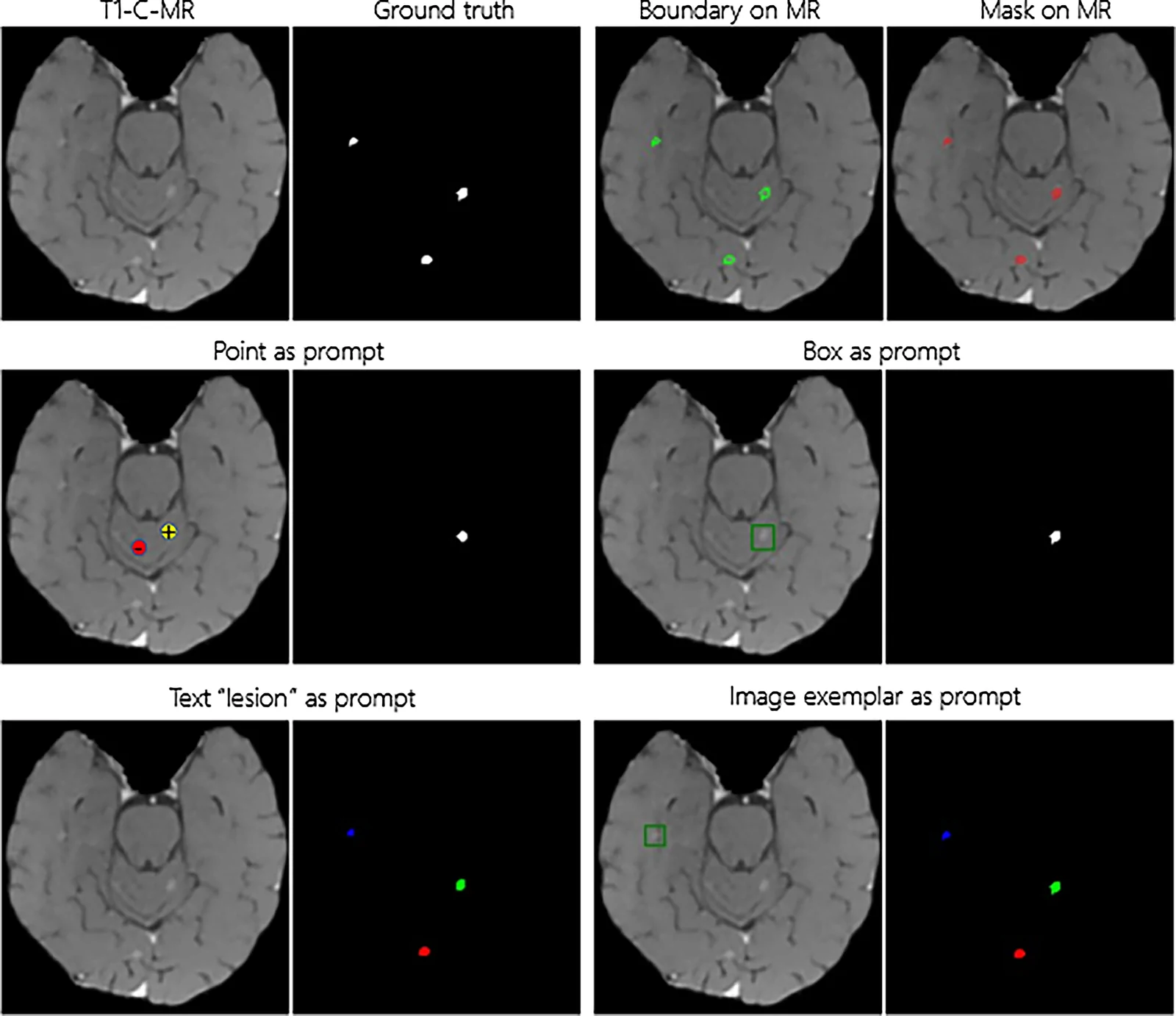
Comparison of geometric-based and concept-based prompts in Segment Anything Model 3. The first row shows a T1-weighted contrast-enhanced magnetic resonance imaging slice, its tumor annotation (three lesions), tumor boundary overlay, and tumor mask overlay. The second row presents geometry-based prompting using point prompts (positive in yellow, negative in red) or a bounding-box prompt, together with the corresponding segmentation results. The third row illustrates concept-based segmentation using either a text prompt (‘lesion’) or an image exemplar (green bounding box) and the resulting prediction masks. Geometry-based prompts yield a single tumor prediction per prompt, whereas concept-based prompting enables simultaneous segmentation of all relevant lesion regions

Despite these advances, existing SAM-based segmentation approaches remain limited to visual prompt-driven interactions and cannot segment object instances using a concept-level prompt, such as text (short phrase) or an image exemplar. In other words, these interactive models require users to manually provide geometric prompts (e.g., points or bounding boxes) for each target instance in an image, which becomes inefficient or impractical in scenarios involving multiple instances. When multiple instances of the same type of object appear in a single image, geometric prompts must be provided individually for each instance. To address this limitation, SAM3 [35] was proposed. It not only retains compatibility with visual prompts, as in SAM and SAM2, but also enables concept-based segmentation, allowing all instances corresponding to a given concept prompt, which is specified by text or an image exemplar, to be segmented simultaneously (Fig. 1).

Recent studies have begun to explore the potential and limitations of SAM3 by analyzing its architectural and functional advantages compared to those of SAM2. Specifically, Sapkota et al. [36] investigated the fundamental differences between SAM2 and SAM3 on natural images to elucidate why SAM2 fails in concept-driven image segmentation. This analysis spanned multiple aspects, including the network architecture, objective functions, prompt types, training datasets, and evaluation metrics. In ref. [37], SAM2 and SAM3 were further compared for the zero-shot segmentation of 3D medical images, with the evaluation restricted to purely geometric prompts and the concept-based mechanism explicitly disabled. The results demonstrated that SAM3 consistently outperformed SAM2 across most medical segmentation tasks, particularly in scenarios involving sparse user annotations or a complex anatomical topology. However, the potential use of SAM3 in lesion segmentation has not been sufficiently explored. Additionally, to improve the domain adaptation capability, SAM3-adapter introduced lightweight adapter modules into the vision encoder blocks and fine-tuned both the adapter sets and the mask decoder of SAM3, resulting in improved segmentation accuracy, robustness, and training efficiency compared with prior SAM-based adaptation strategies [38]. Concurrently, MedSAM3 [39] extended SAM3 to medical image and video segmentation by leveraging open-vocabulary medical concepts as textual prompts. This approach fine-tuned the detector components while freezing the image and text encoders of the original SAM3 architecture using paired medical images and their corresponding labels. The experimental results demonstrated the effectiveness of this strategy in adapting SAM3 to medical imaging tasks.

**Fig. 2:**
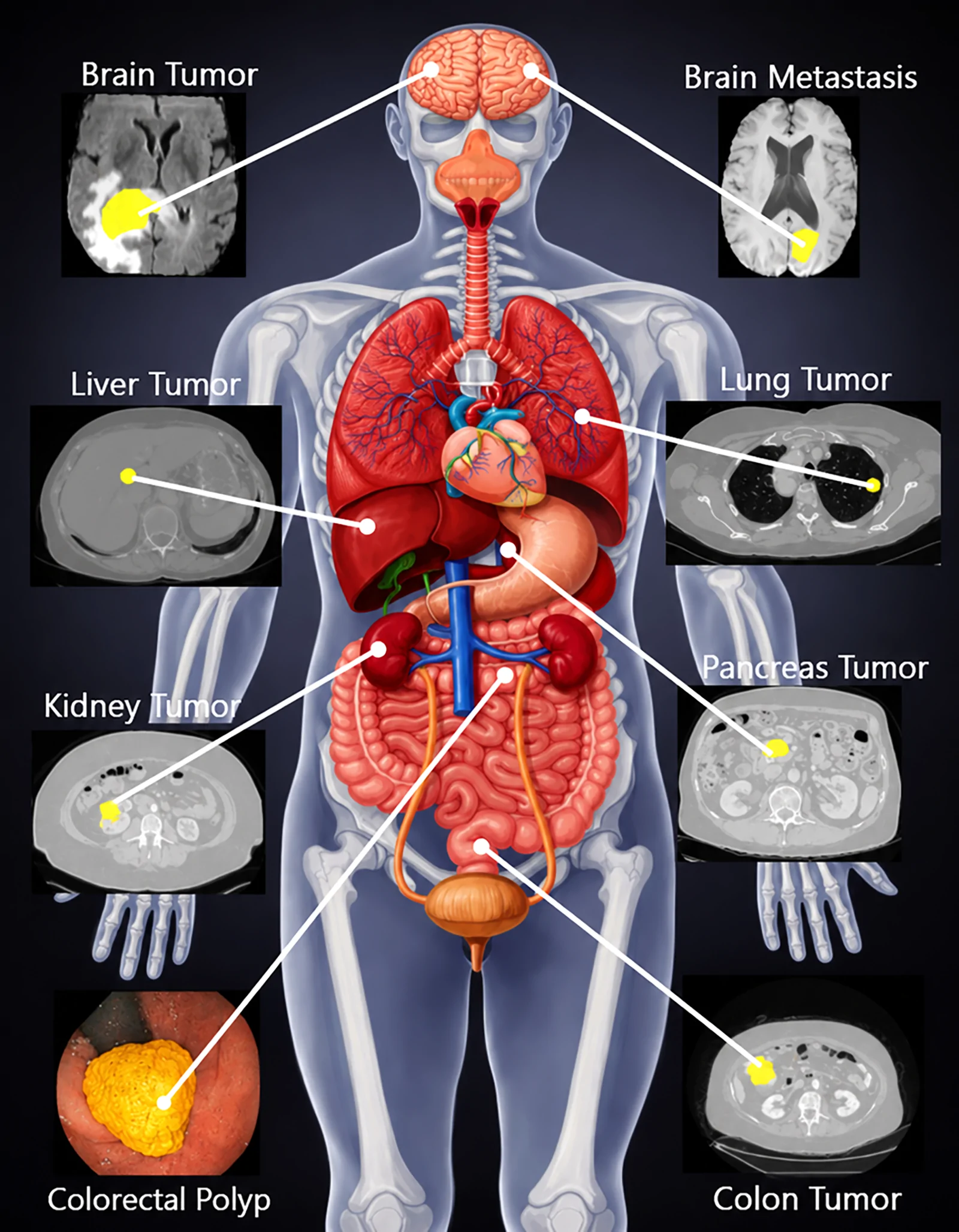
Representative examples of tumor and lesion segmentation overlays across multiple medical imaging modalities and diverse anatomical regions. This study focuses on the systematic evaluation of a single model’s capability to segment heterogeneous lesion types across varied clinical scenarios. The human anatomy illustration is based on a free graphic obtained from Vecteezy.com and was further modified using ChatGPT (https://chatgpt.com/) to enhance visualization

Although a series of studies has been conducted on SAM3, including performance comparisons with SAM2 and fine-tuning using adapters, several important questions remain insufficiently explored in the context of concept-prompt-based (text and/or image exemplars) medical image segmentation: (1) how well SAM3 generalizes to tumor or lesion^1^ segmentation across different anatomical regions, given the substantial variations in anatomical structures, imaging modalities, data distributions, and shape characteristics (Fig. 2); (2) whether integrating prior or additional knowledge (e.g., prediction results from adjacent slices or multimodal imaging concatenation) into the input can effectively improve segmentation performance; and (3) how to fine-tune SAM3 effectively and efficiently for tumor or lesion segmentation in medical images. This study seeks to address these three questions by focusing on lesion segmentation across multiple anatomical regions, prior-knowledge-enhanced input representations, and fine-tuning strategies for SAM3 under concept prompts.

The main contributions of this study are summarized as follows:

1. SAM3 for tumor or lesion segmentation across multiple anatomical regions (brain, lung, liver, pancreas, kidney, skin, etc.) was systematically evaluated using concept-level prompts on MRI, CT, ultrasound, dermoscopy, and endoscopy (Fig. 2).
2. The impact of integrating prior and additional knowledge into SAM3 was investigated, including the use of adjacent prediction masks, multimodal image fusion, and prior annotation.
3. A comprehensive comparison of different fine-tuning strategies for SAM3 was conducted, including partial module fine-tuning, adapter-based fine-tuning, and full-model fine-tuning, under various optimization objectives.

### Lesion segmentation in medical images

Lesion segmentation plays a pivotal role in clinical diagnosis, treatment planning, and disease monitoring. Compared to anatomical structure segmentation, lesion segmentation is considerably more challenging owing to the large variability in lesion size, shape, appearance, and location, as well as the typically low contrast between lesions and surrounding tissues or organs [22, 40, 41]. These factors significantly complicate robust and generalizable model designs across different clinical scenarios^2^.

Over the past decade, substantial progress has been made in lesion segmentation, driven primarily by two key factors. First, a growing number of large-scale publicly available datasets have been curated and released largely through community efforts and challenges. Representative examples include the multimodal brain tumor segmentation (BraTS) benchmark, which evolved from early editions with a limited number of cases to BraTS 2023 [42], comprising 1424 expertly annotated subjects. Similar efforts include the kidney tumor segmentation (KiTS) dataset for kidney tumors [43], the liver tumor segmentation dataset for liver tumors [44], and the medical segmentation decathlon (MSD), which provides ten diverse organ and tumor segmentation tasks. In addition, modality-specific datasets such as breast ultrasound images (BUSIs) [45], ISIC2018 [46], and Kvasir-SEG [47] have advanced research on breast tumors, skin lesions, and polyp segmentation, respectively. Second, significant methodological advances have been made using deep-learning-based segmentation frameworks. CNN architectures, particularly encoder-decoder designs, such as U-Net [3] and its variants [40, 48], have become the dominant paradigm owing to their strong representation capacity and efficiency. More recent approaches have incorporated attention mechanisms, transformer-based modules, and hybrid CNN-transformer architectures, such as TransUNet [10], UNETR++ [14], and Mask2Former [49, 50], to better capture long-range dependencies and contextual information. Despite their success, these methods are typically trained for specific lesion types, anatomical regions, and imaging modalities, limiting their generalizability in heterogeneous clinical environments and often requiring task-specific retraining or extensive fine-tuning.

Motivated by these limitations, recent research has shifted toward foundation models and prompt-driven segmentation frameworks that aim to provide more generalizable and flexible solutions, such as MedSAM [30], LeSAM [51], and Medical SAM2 [52]. Nevertheless, existing SAM-based approaches rely heavily on geometric prompts for each target object and exhibit limited generalization capabilities for lesion segmentation across multiple imaging modalities and whole-body anatomical regions. This study proposes leveraging SAM3 to enable robust concept-driven lesion segmentation across diverse organs, imaging modalities, and clinical scenarios. Thus, the proposed approach aims to bridge the gap between task-specific segmentation models and practical, scalable clinical deployment.

### Concept-based segmentation

With the rapid advancement of LLMs, increasing attention has been devoted to learning joint representations that align the language and visual modalities. A seminal contribution in this direction is contrastive language-image pretraining, which learns semantically interpretable visual representations (visual grounding) through large-scale text supervision, enabling natural language to be mapped onto visual concepts [53]. Building on this paradigm, grounding DINO [54] unifies open-set object detection with grounded pretraining and supports free-form textual prompts as inputs. By further extending this framework, DINO-X [55] integrates multiple perception heads to jointly support object detection, instance segmentation, and object captioning within a unified architecture.

SAM3 adopts pretrained text and vision encoders from the perception encoder (PE) [56] and combines them with a DETR-style detection head and a mask decoder for instance-level segmentation. Similar to SAM2, SAM3 incorporates a tracker with a memory bank to enable object tracking and temporal mask propagation. By leveraging its text and prompt encoders, SAM3 supports concept-driven segmentation, allowing all the object instances corresponding to a given textual description or image exemplar to be simultaneously segmented. This study extended this capability to the more challenging problem of lesion segmentation across multimodal medical images, spanning diverse anatomical regions across the whole body.

### Fine-tuning strategies for foundation models

Training a foundation medical model from scratch is not only computationally expensive, requiring substantial computational resources, but also highly challenging, owing to the time-consuming nature of collecting and annotating large-scale medical images across multiple modalities, which demand significant domain knowledge. Consequently, parameter-efficient fine-tuning (PEFT) methods have been proposed [57].

Generally, PEFT approaches can be broadly categorized into two types based on whether additional parameters are introduced into the foundation model: parameter selection and parameter addition methods. Representative examples of the former include FishMask [58] and BitFit [59], whereas the latter include adapter-based methods [60] and low-rank adaptation techniques [61]. Building upon the parameter-addition strategies, a series of adapter-based approaches has been developed for fine-tuning SAM-based models, such as SAM2-Adapter [62], 3DSAM-Adapter [63], and DD-SAM2 [33]. This study evaluates the effectiveness of different fine-tuning strategies for SAM3 in medical image lesion segmentation tasks.

## Methods

### Model overview

**Fig. 3:**
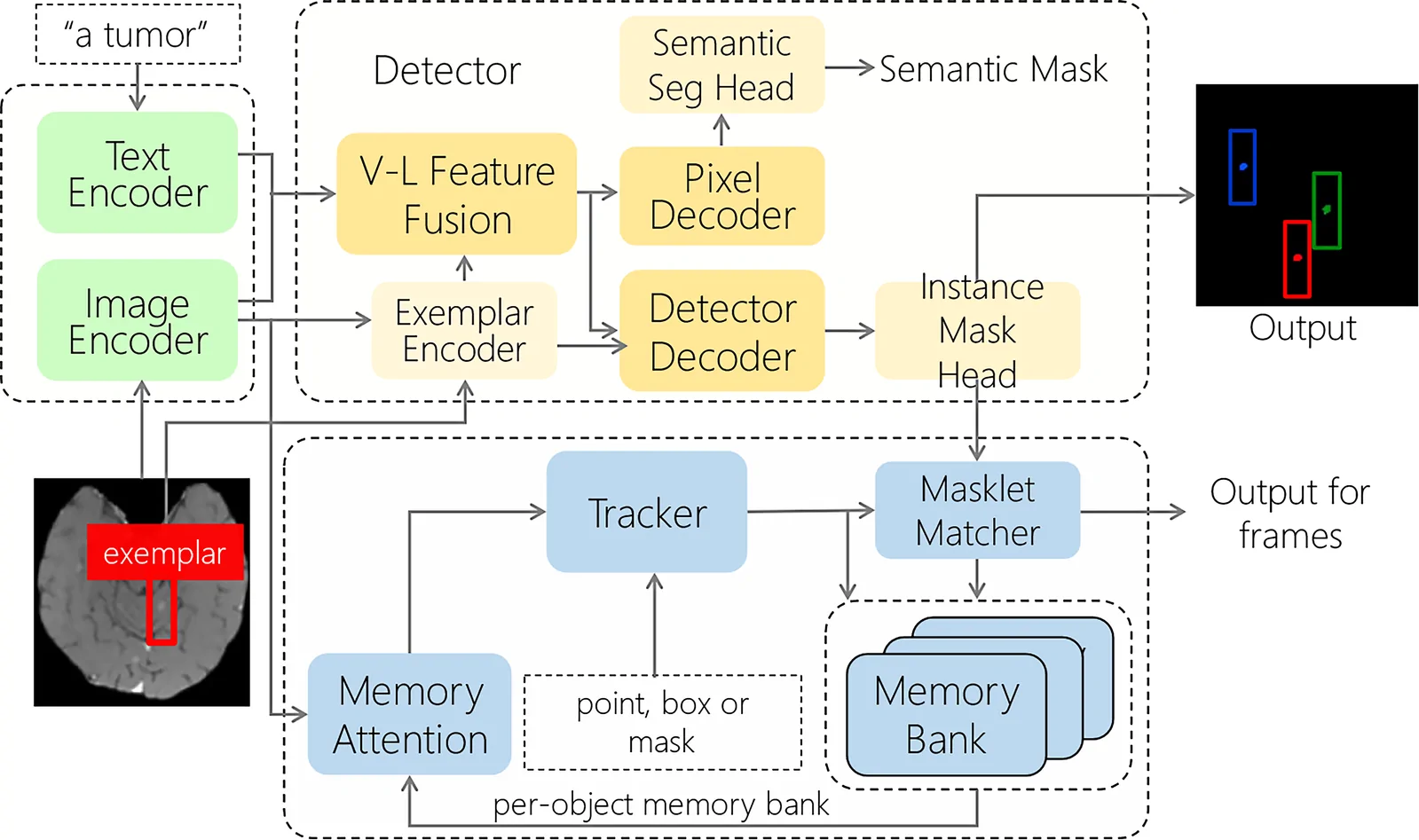
Overview of the Segment Anything Model 3 architecture. The model primarily adopts a dual encoder-decoder transformer framework coupled with a tracker. Specifically, the text encoder and image encoder comprise approximately 450 M and 300 M parameters, respectively, while the detector and tracker together account for about 100 M parameters. This study focuses on concept-based, image-level tumor segmentation; therefore, all object tracking components are disabled, as indicated in blue in the figure. For clarity, several skip connections, such as those linking the visual features from the image encoder to the pixel decoder and the text features from the text encoder to the detector decoder, are omitted to emphasize the core structure of Segment Anything Model 3

SAM3 was designed to support not only geometric prompt-based segmentation (e.g., points, bounding boxes, or masks), as in SAM and SAM2, but also concept prompt-based segmentation using noun phrases or image exemplars. It adopts a dual encoder-decoder transformer architecture in which two encoders independently process visual and textual inputs, whereas two specialized transformer-based decoders are employed for object localization and pixel-wise segmentation, respectively (Fig. 3).

Specifically, SAM3 mainly comprises an image encoder, text encoder, vision-language feature fusion module^3^, pixel decoder, and object detection decoder for image-level, concept prompt-based segmentation. In addition, following SAM2, it is coupled with a tracker and a memory bank to support video object segmentation and tracking (in this study, the video tracking functionality is disabled, and focus is exclusively on image-level segmentation). The image and text encoders are inherited from the PE backbone [56] to extract the image and text embeddings, respectively. The pixel decoder generates dense pixel-level features for semantic mask prediction, whereas the object detection decoder follows a DETR-style design [64], aiming to search for, localize, and identify object instances via learnable detector queries and a presence token. This architecture explicitly decouples the detection, segmentation, and tracking objectives, thereby enabling robust concept-driven and multi-instance segmentation.

### Concept-prompt-based segmentation

This study focuses on concept-based image-level lesion segmentation following a novel promptable concept segmentation paradigm, in which text or image exemplars are provided as prompts. This is in contrast to the promptable visual segmentation (PVS) approach (geometric prompts) employed by SAM and SAM2, although SAM3 also supports PVS segmentation. For text-based prompts, text descriptions specify the instances to be segmented according to a given concept (‘lesions’). The model employs a pretrained text encoder to extract text embeddings, which are then fed into the vision-language feature fusion module and detector decoder, facilitating instance recognition and localization.

In addition, SAM3 supports image exemplars as concept prompts. For example, given a bounding box and a positive binary label (Fig. 3), the model is informed about the target instance to segment. The exemplar encoder processes the geometric information, label class (positive or negative), and region-of-interest-pooled visual features extracted by the image encoder. The detector then integrates these exemplar features to match and identify all instances corresponding to the concept (e.g., the three tumors in Fig. 3), in contrast to the geometric prompts used in the PVS, where each prompt corresponds to only a single segmented object. Furthermore, if text and image exemplars are simultaneously provided, SAM3 can concatenate text embeddings with exemplar feature embeddings to generate unified prompt tokens.

### Prior knowledge enhancement for input

#### Enhancement from preceding prediction masks

On volumetric medical images, lesions typically extend across multiple adjacent slices. Exploiting this spatial continuity as prior knowledge, the authors propose a simple strategy for integrating information from preceding predictions into the current inference step. Specifically, the prediction mask from the preceding slice is concatenated with the current slice and provided as an additional input channel for the model. This strategy supplies coarse localization cues to the current slice. Importantly, the design of presence tokens and learnable detector queries in the object detection decoder enables SAM3 to distinguish whether a lesion exists at the location suggested by the preceding prediction and to accurately match the corresponding region in the current slice when present. Consequently, the model can robustly leverage prior contextual information while avoiding error propagation across slices.

The overall inference procedure can be summarized in the following pseudo code:

**Figure:**
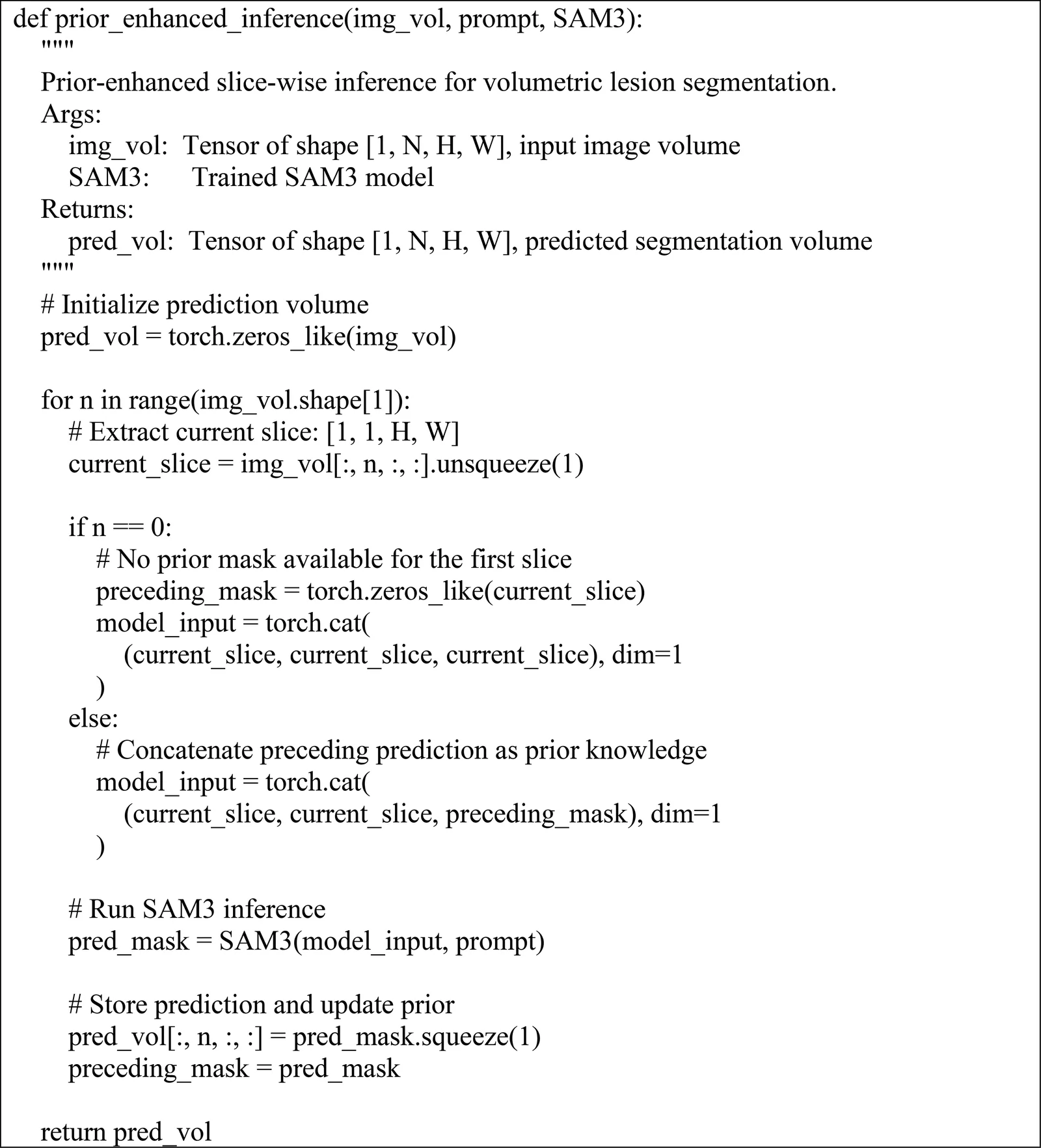

#### Multimodal integration as model inputs

Multimodal imaging offers complementary anatomical and functional information that facilitates more reliable lesion delineation. Therefore, this study explores a multimodal integration strategy in which images from different modalities are concatenated as multichannel inputs to SAM3. This input-level fusion enables the model to exploit cross-modal contrast and structural correlations, potentially improving its robustness to appearance variability and ambiguous boundaries.

#### Enhancement from previous images and annotations

In treatment regimens such as adaptive radiation therapy (ART) [65], prior treatment images and their corresponding annotations provide valuable contextual information regarding lesion location and morphology. To exploit this prior knowledge, the authors incorporated historical annotations and corresponding MR images by concatenating them with the current MR image along the channel dimension. Specifically, for each treatment fraction, the MR image and tumor annotation from the immediately preceding treatment fraction were used as auxiliary inputs to guide the segmentation of the current fraction. Leveraging the most recent prior enabled the model to capture relevant anatomical and morphological characteristics, which is particularly important when the tumor geometry evolves across fractions. This strategy offered coarse spatial guidance that facilitates lesion localization and improves segmentation accuracy. The three prior-based enhancements in the Prior Knowledge Enhancement for Input subsection were evaluated separately without joint evaluation to better characterize the impact of each strategy on the segmentation accuracy.

### Strategies for fine-tuning SAM3

For concept-based image-level segmentation, SAM3 involves three core components: image encoder, text encoder, and detector module, as illustrated in Fig. 3 (highlighted in green and yellow). This study investigated three fine-tuning strategies to adapt SAM3 for lesion segmentation in medical images: full, partial, and adapter-based fine-tuning, as summarized in Table 1. Note that all tracking-related modules are disabled because the focus of the study is on image-level segmentation rather than video object tracking and segmentation.

**Table 1:** Three fine-tuning strategies for Segment Anything Model 3

Mode	Fine-tuning module	Description
Full fine-tuning	Image encoder, text encoder, and detector	End-to-end fine-tuning of all Segment Anything Model 3 components (excluding the tracker).
Partial fine-tuning	Detector	Fine-tuning the entire detector module while freezing the image and text encoders.
Adapter-based fine-tuning	Adapters in the image encoder	Parameter-efficient fine-tuning via lightweight adapter modules inserted into the image encoder, while freezing the original image encoder, the text encoder, and the detector.

**Fig. 4:**
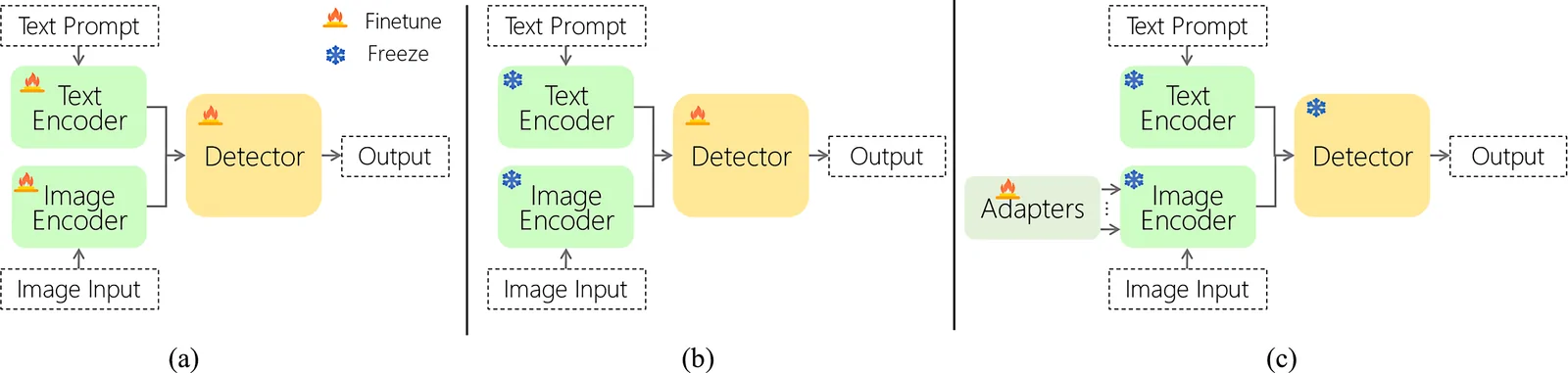
Full (**a**), partial (**b**), and adapter-based (**c**) fine-tuning strategies

For the full fine-tuning mode (Fig. 4a), all parameters of the image encoder, text encoder, and detector were updated during training. To supervise concept-level pixel predictions without relying on explicit instance matching, two pixelwise semantic segmentation losses (focal and Dice losses) were employed. This design enables effective concept segmentation under pixel-level mask supervision. In addition, the detection objective incorporates multiple instance-level loss terms, including cross-entropy loss for query-level classification, alignment loss, and instance-level mask loss, which enforce an accurate spatial alignment between the predicted masks and matched ground-truth instances. This dual-level optimization strategy allows SAM3 to jointly model concept presence, spatial extent, and instance specificity, thereby supporting robust and precise lesion segmentation.

For partial module fine-tuning (Fig. 4b), both the text and image encoders were frozen, and only the detector was fine-tuned. This configuration aims to preserve the general representation capability learned from large-scale pretraining while adapting the task-specific detection head to lesion segmentation. By limiting the number of trainable parameters, this strategy also reduces the risk of overfitting and lowers computational and memory costs. However, freezing the encoders constrains the model’s ability to adapt to domain-specific appearance variations in medical images, which may limit the performance gains. Therefore, an adapter-based fine-tuning approach (Fig. 4c) was adopted as another configuration for adapting SAM3. Following the DD-SAM2 pipeline [33], the adapter modules were inserted into the blocks of the image encoder. During training, only the parameters of the adapter modules were updated, enabling parameter-efficient adaptation while preserving the pretrained backbone weights. When fine-tuning either all parameters or only the detector module, the learning rate was set to 1e-5, the model was trained for 10 epochs, and the AdamW optimizer was used. The final model was selected based on the highest Dice score on the validation set. For adapter-based fine-tuning, the learning rate was increased to 5e-5, and 12 adapter modules were inserted into the 20th - 31st blocks of the image encoder. All other training settings were identical to those described above.

## Results

### Datasets

To evaluate the performance of SAM3 comprehensively across diverse medical imaging modalities, 13 lesion segmentation datasets spanning MRI, CT, dermoscopy, endoscopy, and ultrasound images were curated. These datasets cover a wide range of anatomical regions, including the brain, thorax, abdomen, and skin, and encompass various pathological targets, including brain, lung, liver, pancreatic, and kidney tumors, ischemic stroke lesions, and skin lesions. A detailed summary of the datasets, imaging modalities, target objects, and data splits is presented in Table 2. For evaluations using a single modality as the input, T1-contrast images from BraTS2023-MET and diffusion-weighted imaging (DWI) from ISLES22 were used. For the Mix-Seq-Brain and Mix-Seq-Abdomen datasets, the input imaging modality is randomly selected for each case to evaluate the model’s generalization capability across multiparametric MRI sequences.

**Table 2:** Summary of the medical datasets used for lesion segmentation

Dataset	Modality	Target object	Train	Validation	Test
BraTS2023-MET	T1-C, T1-W, T2-FLAIR, T2-W	Brain metastasis	85	80	73
ISLES22	DWI, ADC	Ischemic stroke lesion	65	60	125
MSD Lung	CT	Lung tumor	18	15	30
MSD Liver	CT	Liver tumor	36	35	60
MSD Pancreas	CT	Pancreas tumor	51	50	180
MSD Colon	CT	Colon tumor	30	30	66
KiTS2019	CT	Kidney tumor	50	50	200
ISIC2018	Dermoscopy	Skin lesion	574	500	1520
Kvasir-SEG	Endoscopy	Polyp	340	340	320
BUSI	Ultrasound	Breast tumor	300	47	300
Mix-Seq-Brain	T1-C, T1-W, T2-FLAIR, T2-W	Brain tumor	-	-	86
One-Seq-Liver	T2-MVXD	Liver tumor	-	-	115
Mix-Seq-Abdomen	T2-MVXD, T2-MVXD-SPIR, T1-3DVaneXD, and T2-FS	Abdomen tumor	-	-	88

- indicates “not applicable”: for Mix-Seq-Brain, One-Seq-Liver, and Mix-Seq-Abdomen, the data are used for test only.

(1) **BraTS2023-MET** [66]. Brain metastases are the most common malignant tumors of the adult central nervous system and arise from primary cancers located elsewhere in the body. BraTS2023-MET is a subtask of the BraTS2023 challenge, designed to segment brain metastases using multimodal MRI, including T1-contrast-enhanced (T1-C), T1-weighted (T1-W), T2-FLAIR (T2-F), and T2-weighted (T2-W) imaging. This study focused on enhancing tumor segmentation and partitioning 238 annotated cases into 85 training, 80 validation, and 73 testing subjects.

(2) **ISLES22** [67]. ISLES22 is a multicenter brain MRI dataset developed for benchmark segmentation algorithms for acute to subacute ischemic stroke lesions. The dataset exhibits substantial variability in lesion size and appearance across DWI, apparent diffusion coefficient (ADC), and FLAIR images. A total of 125 3D cases, including DWI and ADC volumes, with corresponding reference lesion labels, were used for testing.

(3) **MSD Lung** [68]. The MSD Lung dataset is a subset of MSD and consists of 3D CT volumes acquired from patients with non-small cell lung cancer. This task focuses on the segmentation of relatively small lung tumors. In the experiments, 30 testing samples were used.

(4) **MSD Liver** [68]. The MSD Liver dataset contains contrast-enhanced CT scans with annotations for liver tumors. In this study, 60 publicly available annotated cases were tested.

(5) **MSD Pancreas** [68]. The MSD Pancreas dataset comprises CT scans of patients undergoing surgical resection for pancreatic masses. The dataset poses a challenging segmentation problem owing to the severe class imbalance among the background, pancreas, and tumor regions. A total of 180 test samples were utilized, focusing exclusively on pancreatic tumor segmentation.

(6) **MSD Colon** [68]. The MSD Colon dataset is another subset of MSD and focuses on colon cancer segmentation from contrast-enhanced CT images. The dataset includes volumetric scans with pixel-wise annotations of colon tumors, which exhibit considerable variability in size, shape, and location. A total of 66 CT volumes were employed for testing.

(7) **KiTS2019** [43]. The Kidney Tumor Segmentation 2019 (KiTS2019) dataset comprises contrast-enhanced CT scans with expert annotations of renal tumors. Two hundred annotated scans were used as test cases in this study.

(8) **ISIC2018** [46]. The ISIC2018 dataset is part of the International Skin Imaging Collaboration challenge for benchmarking skin lesion segmentation algorithms. It consists of dermoscopic images with expert-annotated lesion masks. In total, 1520 testing images were used in this study.

(9) **Kvasir-SEG** [47]. Kvasir-SEG contains endoscopic images of colorectal polyps accompanied by pixel-wise ground-truth annotations. A total of 320 test samples were used in this study.

(10) **BUSI** [45]. BUSI comprises ultrasound images categorized into normal (133), benign (437), and malignant (210) cases. Normal images were excluded from the study because they do not contain lesion annotations. A total of 300 lesion-bearing images were used as testing samples.

(11) **Mix-Seq-Brain**. The in-house Mix-Seq-Brain dataset consisted of 86 MRI scans collected from 37 patients with brain tumors treated by a 1.5 T MR-LINAC system (Unity, Elekta AB, Stockholm, Sweden), with 1-5 adaptive fractions per patient. The dataset included multiple T1- and T2-weighted MRI sequences, such as T2-FLAIR, T1-weighted, and contrast-enhanced T1-weighted imaging. Expert radiation oncologists manually delineated 97 clinical target volumes (CTVs). Further details are available in ref. [69].

(12) **One-Seq-Liver**. The in-house One-Seq-Liver dataset consisted of 115 MRI scans acquired from 31 patients diagnosed with liver tumors, all obtained using a consistent T2-weighted MultiVane eXtended Dynamic (T2-MVXD) sequence. All patients were treated with a 1.5 T MR-LINAC system, with 1-7 adaptive fractions per patient [57]. In total, 167 internal target volumes were manually delineated by experienced radiation oncologists during routine clinical practice.

(13) **Mix-Seq-Abdomen**. The in-house Mix-Seq-Abdomen dataset comprised 88 fractional MRI scans from 28 MR-LINAC patients with abdominal malignancies, including liver, pancreatic, and renal tumors. Multiple MRI sequences were employed across different treatment fractions, including T2-MVXD, T2-MVXD with fat suppression (T2-MVXD-SPIR), T1-weighted 3D Vane XD (T1-3D-VaneXD), and T2-weighted fat-suppressed turbo spin-echo (T2-FS) imaging. Each patient underwent 2-6 adaptive fractions. In total, 156 ITVs were manually annotated by radiation oncologists.

### Direct evaluation without fine-tuning

#### Comparison between SAM2 and SAM3 using bounding boxes as geometric prompts

To compare the lesion segmentation performance of SAM2 and SAM3 using bounding box-based geometric prompts, tight bounding boxes were extracted directly from the ground-truth masks for each lesion in every image slice. Each bounding box was defined by the top, bottom, left, and right boundaries of the corresponding ground-truth lesion mask. Although this strategy is less practical in real clinical workflows because it requires precise slice-by-slice bounding box annotations, it provides a fair reference for model performance comparison. Table 3 summarizes the segmentation results in terms of Dice, 95th-percentile Hausdorff distance (HD95), and average surface distance (ASD) across all 13 evaluated datasets. These results reflect the upper-bound performances achieved by SAM2 and SAM3 when guided by tight bounding-box prompts.

As presented in Table 3, SAM3 outperformed SAM2 on 10 of the 13 lesion-related datasets in terms of Dice. SAM3 achieved substantial performance gains on ISLES22 (+ 4.41%, in absolute Dice; the same convention is used below), MSD Pancreas (+ 3.04%), and ISIC2018 (+ 5.39%). By contrast, SAM2 exhibited marginal advantages on BraTS2023-MET, MSD Lung, and BUSI, with Dice improvements of < 1%. Overall, these results indicate that SAM3 demonstrates superior lesion segmentation capability compared with SAM2 when evaluated under conventional geometric prompting conditions.

**Table 3:** Comparison of segmentation performance between SAM2 and SAM3, using bounding box prompts for every lesion across multiple medical datasets

Dataset	Method	Dice (%)↑	HD95 (voxel)↓	ASD (voxel)↓
BraTS2023-MET	SAM2	88.52	2.18	0.65
	SAM3	87.92	1.45	0.54
ISLES22	SAM2	81.26	1.54	0.35
	SAM3	85.67	1.15	0.31
MSD Lung	SAM2	87.01	2.35	0.63
	SAM3	86.90	2.38	0.64
MSD Liver	SAM2	84.37	4.21	1.33
	SAM3	86.04	3.24	1.03
MSD Pancreas	SAM2	83.88	10.12	2.39
	SAM3	86.92	6.13	1.46
MSD Colon	SAM2	82.52	2.98	0.69
	SAM3	83.89	2.96	0.60
KiTS2019	SAM2	90.23	4.00	1.10
	SAM3	91.94	3.02	0.77
ISIC2018	SAM2	85.43	246.11	95.00
	SAM3	90.82	119.78	46.25
Kvasir-SEG	SAM2	94.33	27.78	8.66
	SAM3	94.52	21.82	7.22
BUSI	SAM2	87.49	25.94	9.16
	SAM3	87.41	26.79	9.92
Mix-Seq-Brain	SAM2	76.15	17.05	4.65
	SAM3	77.56	8.45	2.74
One-Seq-Liver	SAM2	82.19	4.11	1.17
	SAM3	83.28	3.90	1.06
Mix-Seq-Abdomen	SAM2	83.96	3.51	1.02
	SAM3	85.62	3.07	0.87

↑ indicates higher is better; ↓ indicates lower is better. HD95: 95th-percentile Hausdorff distance; ASD: Average surface distance

### Comparison of fully supervised learning methods

To systematically evaluate model performance, supplementary experiments were carried out comparing SAM2 and SAM3 against six mainstream fully supervised segmentation architectures–SegNet [70], U-Net, Attention U-Net [71], UNet++ [4], SegResNet [72], and SegFormer [73]–across three public medical image segmentation benchmarks: Kvasir-SEG, ISIC2018, and BUSI.

To guarantee a fair and rigorous comparison, all supervised baselines were trained and tested using identical data splits as Table 2. All the models were optimized using the Adam optimizer with an initial learning rate of 1e-4 and a weight decay of 1e-5. Considering the varying dataset scales, the number of training epochs was set to 500 for Kvasir-SEG, 200 for ISIC2018, and 500 for BUSI. Moreover, all models were evaluated based on the original image resolution following the unified evaluation protocol for SAM2 and SAM3.

The quantitative results for all the datasets are summarized in Table 4. The experimental results demonstrated that under bounding box prompts, both SAM2 and SAM3 achieved competitive or superior performance compared to the representative fully supervised segmentation baselines on all three medical datasets. SAM3 exhibited the best overall performance.

**Table 4:** Comparison with fully supervised learning methods on Kvasir-SEG, ISIC2018, and BUSI

Dataset	Method	Dice (%)↑	HD95 (voxel)↓	ASD (voxel)↓
Kvasir-SEG	SAM2	94.33	27.78	8.66
	SAM3	94.52	21.82	7.22
	SegNet	53.67	164.44	62.87
	U-Net	70.62	152.88	45.13
	Attention-UNet	71.14	117.92	34.49
	UNet++	67.57	148.32	43.82
	SegResNet	69.33	142.36	43.2
	SegFormer	54.15	156.24	54.04
ISIC2018	SAM2	85.43	246.11	95.00
	SAM3	90.82	119.78	46.25
	SegNet	83.55	262.35	77.63
	U-Net	83.54	272.96	84.71
	Attention-UNet	82.84	269.71	89.39
	UNet++	83.56	293.82	85.66
	SegResNet	85.97	197.47	60.13
	SegFormer	84.72	218.75	81.2
BUSI	SAM2	87.49	25.94	9.16
	SAM3	87.41	26.79	9.92
	SegNet	57.99	130.03	57.80
	U-Net	64.25	151.93	53.06
	Attention-UNet	66.03	111.53	41.13
	UNet++	66.15	134.32	49.57
	SegResNet	73.43	91.00	28.01
	SegFormer	67.18	114.76	34.83

↑ indicates higher is better; ↓ indicates lower is better. HD95: 95th-percentile Hausdorff distance; ASD: Average surface distance

### Performance comparison between concept-based prompts for SAM3

In this setting, the performance of SAM3 under concept-based prompting was evaluated. Specifically, three prompting strategies were compared: pure text (T), single-image exemplar (E), and a combination of text and image exemplars (T + E). For 2D single-lesion datasets such as ISIC2018 and Kvasir-SEG, E and T + E are still viewed as concept-based prompting strategies, even if the exemplar is drawn from the same test lesion. Because each image contained only a single lesion, the lesion itself was used as an exemplar to guide the final segmentation. Compared to a geometric prompt, such as a same-location bounding box, guidance from the exemplar anchors the features of the selected region.

#### Comparison with various concept-based prompts

In addition, the generic term ‘lesion’ is adopted as the text prompt for all datasets unless otherwise specified. For the image exemplar, a tight bounding box enclosing a lesion was provided to SAM3 as a visual concept prompt, enabling the model to identify all lesions with similar semantic characteristics on the target slice. In the current evaluation protocol, an image exemplar was randomly selected from the same test slice during inference. Specifically, one lesion instance on the target slice was randomly chosen, and its tight bounding box was used to crop the exemplar region that served as the concept prompt, always drawn from the same slice rather than from another slice, volume, or patient. Consequently, the proposed framework assumes prior knowledge of the location of at least one lesion in each test slice. In all the experiments, the random seed was fixed to 2024 for Python, NumPy, PyTorch, and all related data loading and training processes to ensure reproducibility. 

Table 5 summarizes the segmentation performance of the three concept-based prompting strategies in terms of Dice, HD95, and ASD. The occurrence of NaN (for HD95) and Inf (for ASD) reflects the existence of failed segmentation cases in the text-only prompting setting, where the predicted mask or ground-truth mask contains no valid foreground pixels, making the corresponding surface-distance metrics undefined.

**Table 5:** Segmentation performance of SAM3 using concept-based prompts for volume medical datasets: text (T), image exemplar (E), and combined text and image exemplar (T + E)

Dataset	Prompt	Dice (%)↑	HD95 (voxel)↓	ASD (voxel)↓
BraTS2023-MET	T	0.69	NaN	Inf
	E	74.39	12.96	4.68
	T + E	77.29	4.24	1.06
ISLES22-DWI	T	42.46	NaN	Inf
	E	68.44	7.29	1.96
	T + E	64.94	6.27	1.55
MSD Lung	T	62.36	NaN	Inf
	E	83.90	29.34	9.63
	T + E	86.97	2.28	0.76
MSD Liver	T	23.95	NaN	Inf
	E	75.91	24.5	9.84
	T + E	77.45	6.29	1.84
MSD Pancreas	T	3.96	NaN	Inf
	E	82.25	23.87	7.17
	T + E	84.97	4.74	1.31
MSD Colon	T	9.17	NaN	Inf
	E	81.87	18.88	6.39
	T + E	83.86	2.39	0.43
KiTS2019	T	8.71	NaN	Inf
	E	79.30	82.89	37.99
	T + E	90.84	8.81	2.12
Mix-Seq-Brain	T	11.43	NaN	Inf
	E	73.03	13.84	3.86
	T + E	74.29	9.28	3.00
One-Seq-Liver	T	3.92	NaN	Inf
	E	72.93	26.48	8.52
	T + E	76.28	5.42	1.60
Mix-Seq-Abdomen	T	8.94	NaN	Inf
	E	79.17	9.76	2.38
	T + E	80.01	5.73	1.40

↑ indicates higher is better; ↓ indicates lower is better. NaN denotes not a number, and Inf denotes infinity. HD95: 95th-percentile Hausdorff distance; ASD: Average surface distance

As presented in Table 5, text-only prompts (T) consistently yielded the poorest segmentation performance across all evaluated datasets, indicating that relying solely on textual descriptions was insufficient for accurate lesion recognition and delineation. For instance, the Dice scores obtained with text-only prompts were extremely low for several datasets, including 0.69% for BraTS2023-MET, 3.96% for MSD Pancreas, 9.17% for MSD Colon, and 8.71% for KiTS2019, highlighting the inherent difficulty of localizing lesions without visual guidance. In contrast, image-exemplar-based prompting (E) substantially improved the segmentation performance across all datasets. Moreover, the combined use of text and image exemplars (T + E) generally achieved the best or near-best performance, outperforming image exemplars in most cases. Specifically, the Dice score improved by 2.90%, 3.07%, 2.72%, and 11.54% for the BraTS2023-MET, MSD Lung, MSD Pancreas, and KiTS2019 datasets, respectively. The only exception was observed for ischemic stroke lesion segmentation on the ISLES22-DWI dataset, where adding text prompts led to a slight degradation in Dice.

Despite this decrease in overlap accuracy, the T + E prompting strategy consistently improved the surface-based metrics (HD95 and ASD) across all datasets, suggesting that textual prompts contribute to more accurate boundary delineation. Overall, these results demonstrate that visual exemplars play a critical role in concept-based lesion segmentation, whereas textual prompts provide complementary semantic guidance when used in conjunction with image exemplars.

#### Comparison with various text prompts

An experiment was conducted to evaluate the effects of different text prompts on the MSD Lung dataset. Specifically, a diverse set of prompts was evaluated, ranging from simple descriptions (e.g., ‘lesion’ and “lung lesion”) to more specific clinical and radiological expressions (e.g., “malignant lung nodule,” “primary lung carcinoma lesion,” and “small focal lung tumor lesion”). A complete list of the evaluated prompting strategies and their corresponding quantitative results is presented in Table 6.

**Table 6:** Comparison with various text prompts on MSD Lung

Text prompt	Dice (%)↑
“lesion”	62.36
“lesion in lung”	62.45
“find lesion in lung”	1.04
“tumor”	0.00
“lung tumor”	6.18
“pulmonary tumor”	0.24
“malignant lung nodule”	1.73
“lung lesion”	61.63
“pulmonary lesion”	65.34
“solid lung mass”	0.06
“intrapulmonary neoplasm”	3.21
“primary lung carcinoma lesion”	61.70
“focal abnormal mass inside lung lobe”	0.00
“malignant intrapulmonary soft tissue lesion”	58.58
“localized lung tumor surrounded by normal lung tissue”	0.00
“abnormal neoplastic lesion in lung parenchyma”	8.91
“tiny malignant pulmonary nodule in lung”	0.25
“small focal lung tumor lesion”	62.08
“minute intrapulmonary neoplastic mass”	0.00
“peripheral lung tumor adjacent to pleura”	3.23
“cavitary malignant pulmonary lesion”	56.17
“isolated malignant lesion within lung lobe”	4.62

↑ indicates higher is better

The experimental results reveal that the segmentation performance is highly sensitive to the choice of text prompts. For example, the prompt ‘lesion’ achieves a Dice score of 62.36%, whereas the semantically similar prompt ‘tumor’ yields a 0.00% Dice. Likewise, “lung lesion” achieves a 61.63% Dice, while “lung tumor” obtains only a 6.18% Dice. Even clinically meaningful descriptions, such as “malignant lung nodule” or “solid lung mass,” result in poor performance. These results demonstrate that the current SAM3 text encoder has limited robustness against semantically similar medical expressions.

This limitation may arise because the original text encoder was primarily trained on natural image-text pairs and lacked sufficient medical domain knowledge to effectively distinguish fine-grained clinical terminology and align it with medical image representations. Consequently, although text prompting is supported by SAM3, its effectiveness in medical image segmentation remains highly dependent on the specific wording of the prompts. Improving the medical understanding of text encoders through domain-specific adaptation or multimodal alignment is an important direction for future research.

### Prior-driven input augmentation

#### Input augmentation with preceding slices’ prediction masks

Table 7 presents the results of incorporating prediction masks from adjacent preceding slices as an additional input channel. This strategy aims to provide coarse localization priors, under the assumption that lesions typically span multiple adjacent slices. As presented in Table 7, integrating the previous prediction mask leads to Dice improvements on several datasets, for instance, BraTS2023-MET, Mix-Seq-Brain, and Mix-Seq-Abdomen, demonstrating that even simple mask-based priors can be beneficial for lesion segmentation.

However, the effectiveness of this strategy was not consistent across all datasets. For example, performance degradation was observed on MSD Lung, and metric trade-offs were evident on Mix-Seq-Brain, where Dice improved substantially, while the surface-based metric HD95 deteriorated. These results suggest that naïvely concatenating preceding prediction masks may introduce noisy or misleading spatial cues, particularly when lesions exhibit discontinuous appearances across slices, significant shape variations, or prediction errors propagating from preceding slices. More robust strategies such as confidence-aware mask fusion or selective gating mechanisms may be necessary to fully exploit mask-based priors while avoiding error accumulation and false-positive propagation.

**Table 7:** Quantitative comparison of segmentation performance with and without using preceding slices’ prediction masks as prior for four public and three in-house datasets

Dataset	Preceding Slice’s prediction mask	Dice (%)↑	HD95 (voxel)↓	ASD (voxel)↓
BraTS2023-MET	w/o	77.29	4.24	1.06
	w	78.11	3.20	0.88
MSD Lung	w/o	86.97	2.28	0.76
	w	84.15	3.23	1.02
KiTS2019	w/o	90.84	8.81	2.12
	w	90.33	4.62	0.86
Mix-Seq-Brain	w/o	74.29	9.28	3.00
	w	78.24	9.79	2.86
One-Seq-Liver	w/o	76.28	5.42	1.60
	w	76.29	10.56	1.05
Mix-Seq-Abdomen	w/o	80.01	5.73	1.40
	w	80.74	8.35	1.15

Results are reported for models using text and image exemplar prompts. ↑ indicates higher is better; ↓ indicates lower is better. HD95: 95th-percentile Hausdorff distance; ASD: Average surface distance

#### Input augmentation with multimodal images

To assess the effectiveness of multimodal input augmentation, the proposed approach was evaluated using two multimodal datasets: BraTS2023-MET and ISLES22. As summarized in Table 8, the incorporation of multiple imaging modalities generally leads to improved segmentation performance, indicating that complementary modality-specific information is beneficial for lesion delineation.

On the BraTS2023-MET dataset, progressively adding imaging modalities consistently improved performance. In particular, combining T1-C, T2-F, and T2-W images yielded a 2.78% absolute Dice improvement compared with using only the T1-C modality. These results demonstrate that multimodal fusion effectively enriches the lesion-related contextual information and enhances the segmentation robustness. For the ISLES22 dataset, while single-modal performance varied across DWI and ADC inputs, their joint use resulted in improved surface-based metrics (HD95 and ASD), highlighting the complementary nature of diffusion-derived images. However, the Dice of the combined inputs improved modestly over the ADC-only results, whereas it was inferior to the DWI-only inputs. This suggests that the modality relevance and quality play critical roles in determining the effectiveness of multimodal fusion. Overall, these findings indicate that input-level multimodal concatenation is a simple yet potentially effective strategy for improving lesion segmentation performance, although its benefits may be dataset- and modality-dependent.

**Table 8:** Quantitative evaluation of multimodal input augmentation on BraTS2023-MET and ISLES22 datasets

Dataset	Input	Dice (%)↑	HD95 (voxel)↓	ASD (voxel)↓
BraTS2023-MET	T1-C	77.29	4.24	1.06
	T1-C + T2-F	77.82	3.75	0.99
	T1-C + T1-W	77.53	3.55	0.98
	T1-C + T2-W	78.61	3.22	0.89
	T1-C + T2-F+T1-W	79.25	3.80	1.08
	T1-C + T2-F+T2-W	80.07	4.03	1.08
ISLES22	DWI	64.94	6.27	1.55
	ADC	58.03	6.24	1.43
	DWI + ADC	60.09	5.26	0.79

Results are reported for models using text and image exemplar prompts. ↑ indicates higher is better; ↓ indicates lower is better. HD95: 95th-percentile Hausdorff distance; ASD: Average surface distance

#### Input augmentation by using prior images or annotations

Treatment regimens such as ART involve repeated imaging and segmentation of the same patient across multiple sequential treatment fractions, where prior knowledge is usually available to guide each patient-specific segmentation task. To leverage this information, three in-house datasets were used that included both current and prior MR images, along with their corresponding tumor annotations from previous treatment sessions. As described in the Enhancement from Previous Images and Annotations subsection, the current MR image was supplemented with prior MR images and their annotations, resulting in a three-channel input that encodes both the temporal and anatomical contexts for segmentation. The quantitative results are summarized in Table 9. Incorporating prior MR information yielded consistent performance gains, achieving absolute Dice improvements of 10.79%, 7.87%, and 4.47% on the Mix-Seq-Brain, One-Seq-Liver, and Mix-Seq-Abdomen datasets, respectively. These findings indicated that the integration of previous MR images and annotations effectively enhances SAM3’s performance for lesion segmentation in the ART setting.

**Table 9:** Segmentation performance of SAM3 with and without prior MR images and annotations as guidance. Results are reported for models using text and image exemplar prompts

Dataset	Prior image/annotation-guided	Dice (%)↑	HD95 (voxel)↓	ASD (voxel)↓
Mix-Seq-Brain	No	74.29	9.28	3.00
	Yes	85.08	6.89	1.97
One-Seq-Liver	No	76.28	5.42	1.60
	Yes	84.15	2.89	0.74
Mix-Seq-Abdomen	No	80.01	5.73	1.40
	Yes	84.48	3.03	0.82

↑ indicates higher is better; ↓ indicates lower is better. HD95: 95th-percentile Hausdorff distance; ASD: Average surface distance

### Evaluation of fine-tuning strategies

To evaluate the effectiveness of fine-tuning, SAM3 was fine-tuned using the BraTS2023-MET dataset. Three fine-tuning strategies were investigated: fine-tuning all model parameters, fine-tuning only the detector module, and fine-tuning only the additional adapter modules. An overview of these strategies is presented in Fig. 4.

Table 10 presents a quantitative comparison of different fine-tuning strategies on the BraTS2023-MET dataset. Among all approaches, adapter-based fine-tuning achieved the best overall performance, yielding the highest Dice score (82.96%) and the lowest ASD (0.9). In contrast, fine-tuning all the parameters or only the detector module provided limited or inconsistent improvements compared to the baseline without fine-tuning. These results highlight the effectiveness of parameter-efficient adapter-based fine-tuning for lesion segmentation on the BraTS2023-MET dataset.

**Table 10:** Comparison between different fine-tuning strategies on the BraTS2023-MET dataset

FT module	Dice (%)↑	HD95 (voxel)↓	ASD (voxel)↓
None	77.29	4.24	1.06
All	79.16	6.81	1.45
Detector	76.42	6.22	1.03
Adapter	82.96	5.42	0.90

↑ indicates higher is better; ↓ indicates lower is better. HD95: 95th-percentile Hausdorff distance; ASD: Average surface distance

To further evaluate the effectiveness of the adapter-based fine-tuning strategy for the lesion-focused SAM3 model (LeSAM3), additional training and testing were conducted on the combined set of five public datasets. Consistent with the training protocol, the T + E inference strategy (text prompt ‘lesion’ paired with image exemplars) was adopted. A quantitative comparison with the original SAM3 is presented in Table 11.

The results indicate that simply fine-tuning SAM3 using a unified adapter-based paradigm on aggregated multisource datasets does not consistently improve performance across all tasks. Specifically, LeSAM3 achieved higher Dice scores on four of the five datasets, BraTS2023-MET, ISLES22, ISIC2018, and Kvasir-SEG, but exhibited degraded performance on the MSD Pancreas dataset. Moreover, improvements in Dice are not always accompanied by consistent gains in boundary-based metrics such as HD95 and ASD, suggesting that a unified adaptation strategy may struggle to capture the substantial heterogeneity among different lesion types and imaging modalities.

These findings suggest that adapter-based fine-tuning alone is insufficient for achieving robust cross-modality generalization in universal lesion segmentation. Future studies should explore more expressive optimization paradigms, including increasing the number of learnable parameters, designing modality-aware adaptation modules, and developing more effective PEFT strategies to better exploit the representation capability of SAM3 across diverse medical imaging scenarios.

**Table 11:** Comparison of original SAM3 and LeSAM3 (fine-tuned from SAM3 with adapter)

Dataset	Method	Dice (%)↑	HD95 (voxel)↓	ASD (voxel)↓
BraTS2023-MET	SAM3	77.29	4.24	1.06
	LeSAM3	81.77	4.88	0.72
ISLES22	SAM3	64.94	6.27	1.55
	LeSAM3	65.14	6.43	1.74
MSD Pancreas	SAM3	84.97	4.74	1.31
	LeSAM3	84.47	7.02	1.78
ISIC2018	SAM3	73.65	N/A	N/A
	LeSAM3	82.93	N/A	N/A
Kvasir-SEG	SAM3	76.51	N/A	N/A
	LeSAM3	87.72	55.97	15.36

↑ indicates higher is better; ↓ indicates lower is better. HD95: 95th-percentile Hausdorff distance; ASD: Average surface distance

### Visual results

#### Visual results for SAM2 and SAM3 using bounding box prompts

**Fig. 5:**
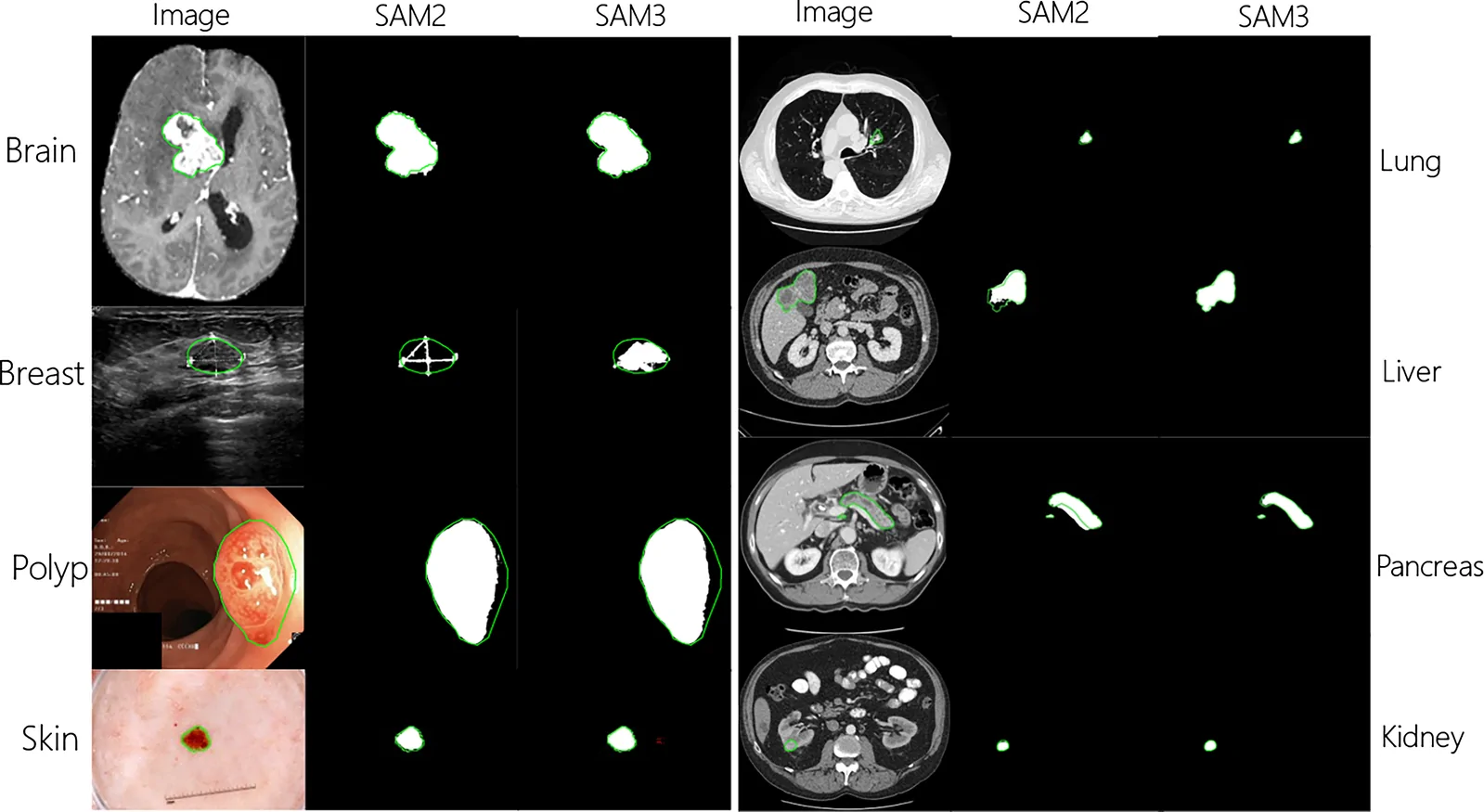
Visualization of lesion segmentation results obtained with Segment Anything Model 2 and Segment Anything Model 3 using bounding boxes as prompts. The green contours indicate the ground truth annotations, and the white masks represent the predicted segmentation results. The same color convention is used in the following figures

Figure 5 presents the representative lesion segmentation results obtained using SAM2 and SAM3 across multiple datasets, including the brain, breast, polyp, skin, lung, liver, pancreas, and kidney, where tight bounding boxes are used as geometric prompts. Overall, both models demonstrated comparable segmentation performances for most samples, indicating a similar generalization ability across different anatomical structures and imaging modalities. However, SAM3 exhibited an improved contextual understanding in several challenging cases, as reflected in the breast and pancreatic examples shown in Fig. 5. In these instances, SAM3 produced segmentations that better aligned with lesion boundaries and the surrounding anatomical context, suggesting that the model benefited from enhanced feature representation and a stronger global reasoning capability than SAM2.

**Fig. 6:**
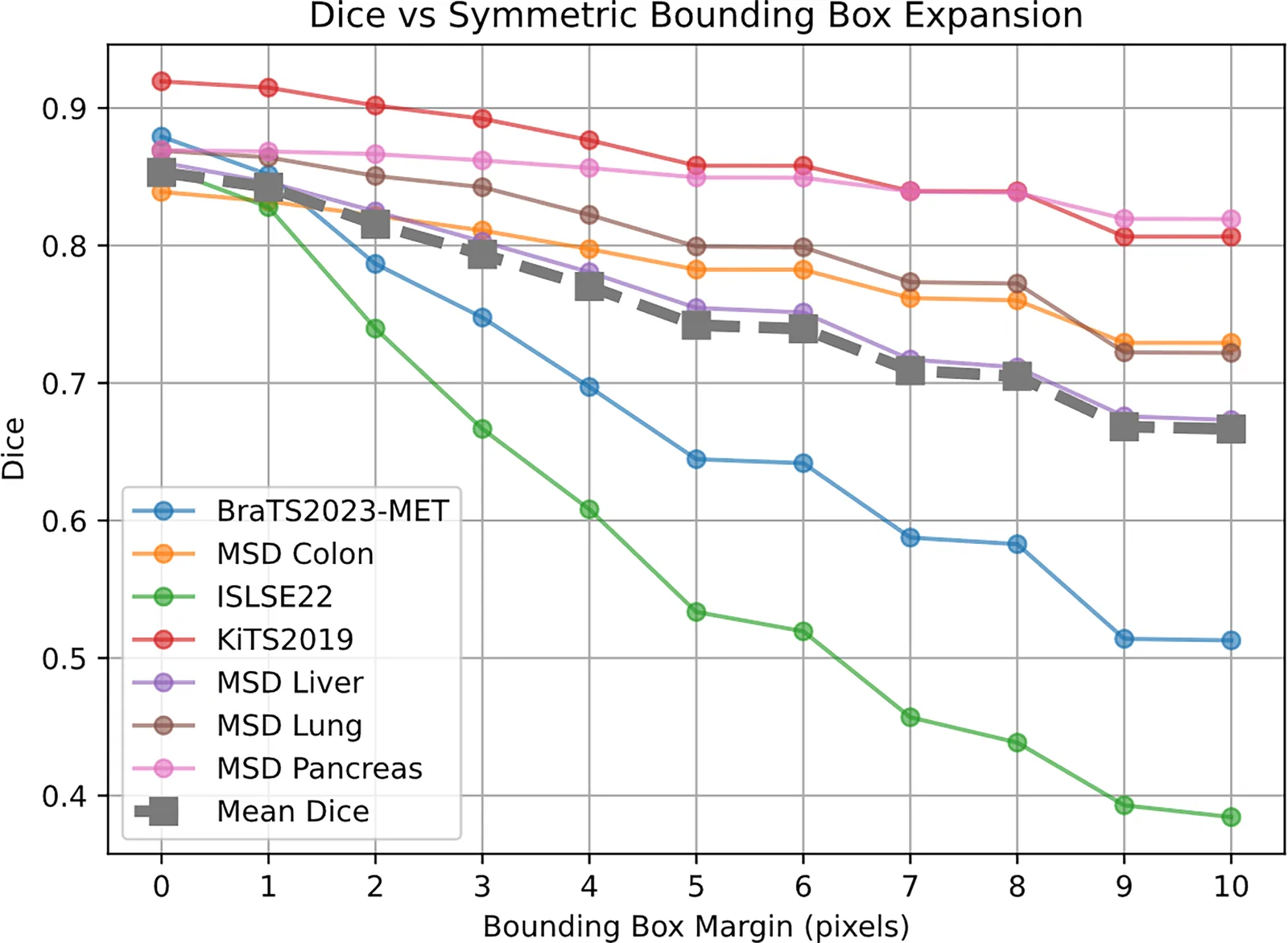
Degradation of Dice performance with progressively enlarged bounding box margins on seven public lesion segmentation datasets using Segment Anything Model 3. The gray dashed line indicates the mean Dice across all seven datasets

Additionally, experiments were conducted with progressively expanded bounding box margins as a prompt quality sensitivity analysis, and the results are summarized in Fig. 6. SAM3 attained a competitive average Dice of 0.85 with tight bounding boxes (gray baseline). Nevertheless, the segmentation performance dropped gradually with expanding box margins; expanding the bounding box by 10 pixels uniformly on the four sides reduced the average Dice metric to approximately 0.68. This result shows that SAM3 is sensitive to the bounding box quality, which further justifies the development of additional concept-driven prompts.

#### Visual results for SAM3 using concept-based prompts

**Fig. 7:**
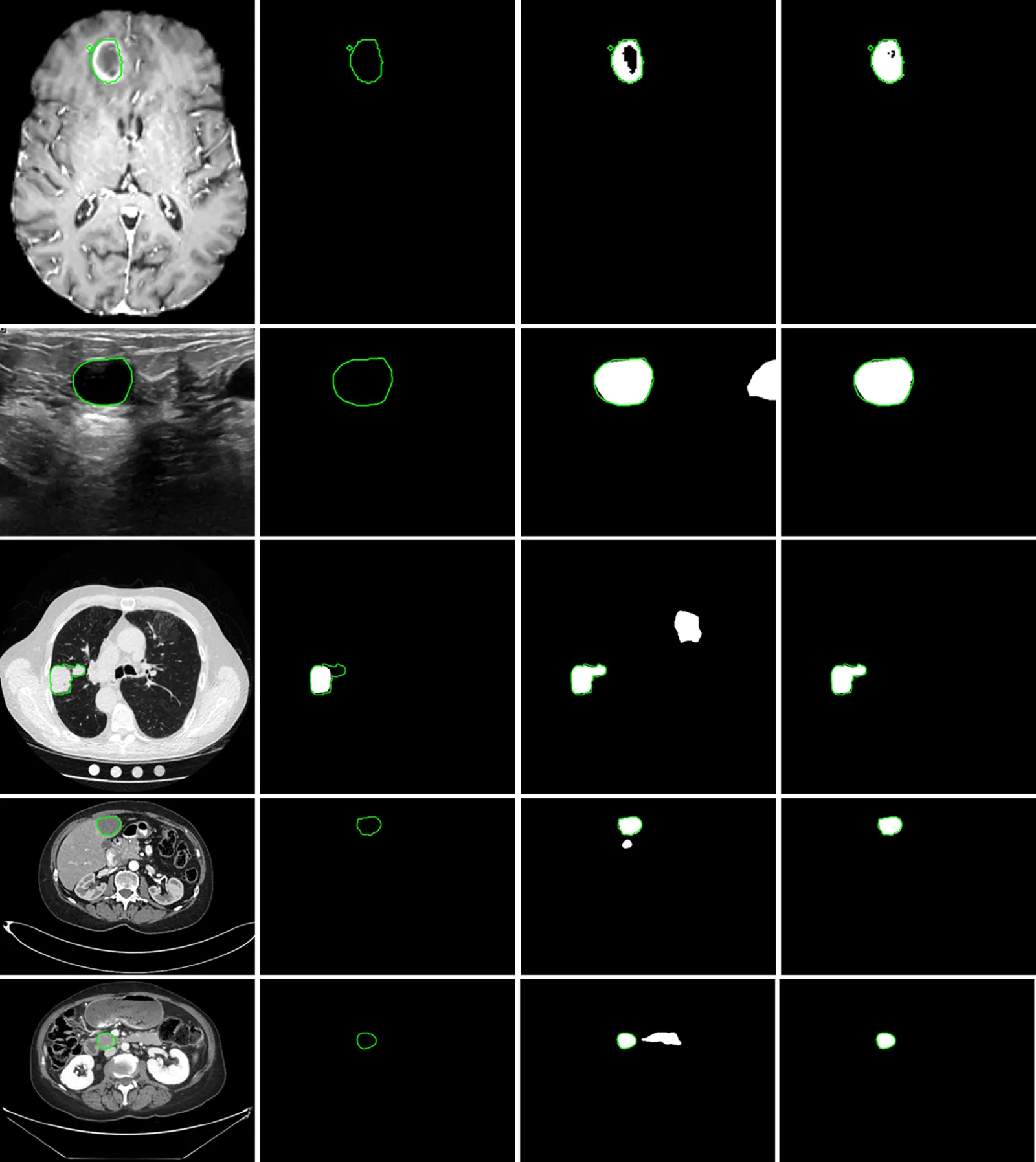
Visualization of lesion segmentation results in the brain, breast, lung, liver, and pancreas using different prompt types, including text (2nd column), image exemplar (3rd column), and their combination (4th column)

Figure 7 demonstrates visual results of SAM3 for lesion segmentation using three types of prompts: text description (‘lesion’), image exemplar, and the combination of text and image exemplar. Examples from the brain, breast, lung, liver, and pancreatic datasets are shown. The results demonstrate that (1) using only text prompts yields the poorest performance, as several lesions cannot be correctly detected in multiple cases, as shown in Fig. 7; (2) using image exemplars significantly improves segmentation accuracy; however, it tends to produce over-segmentation in some cases, such as the breast and lung examples; and (3) combining text and image exemplar prompts achieves the best overall performance, producing more accurate lesion localization and boundaries across different organs.

#### Visual results for SAM3 with and without previous images and annotations as prior knowledge

**Fig. 8:**
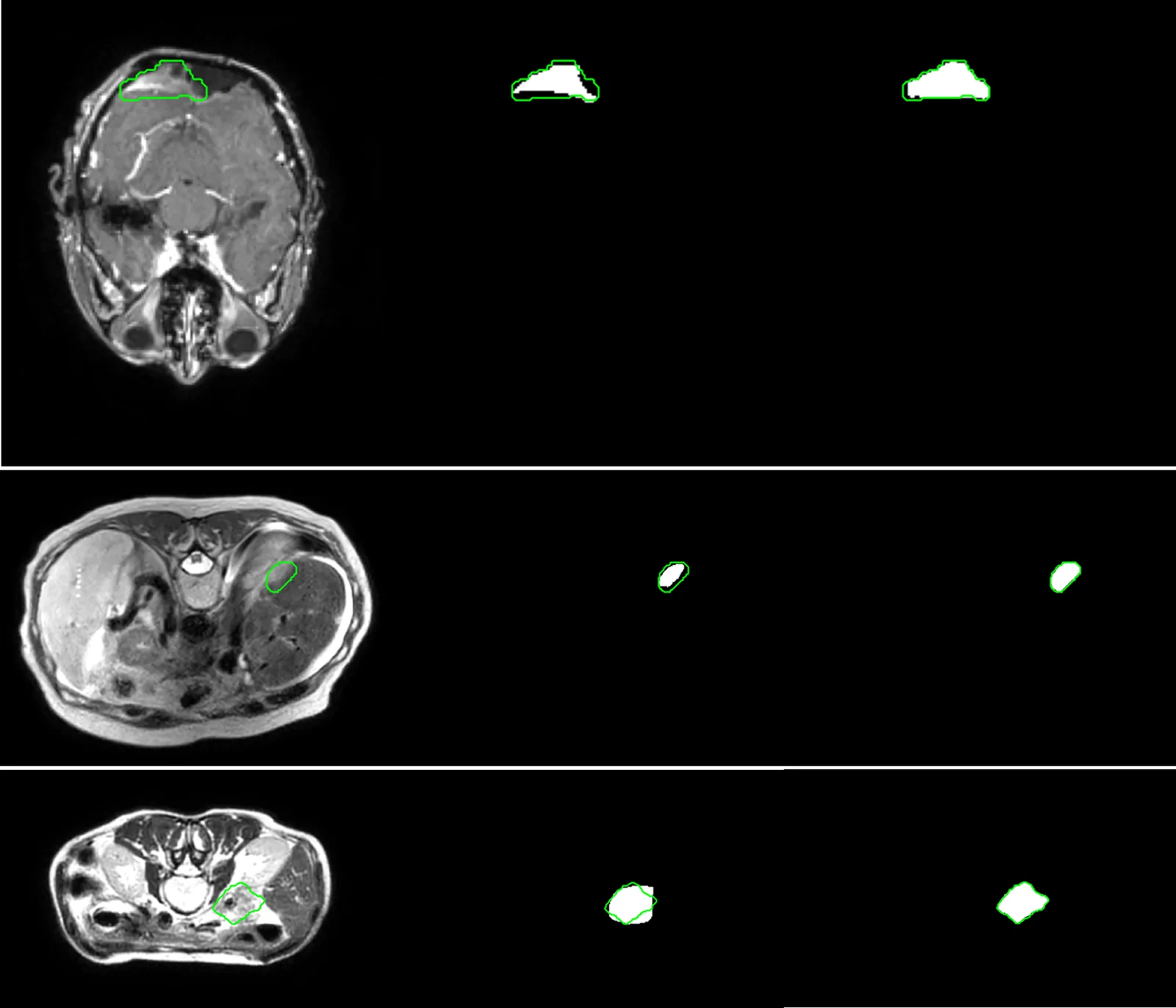
Visual segmentation results without prior image and annotation knowledge (2nd column) and with prior knowledge (3rd column)

Figure 8 presents visual results on the in-house ART datasets, including Mix-Seq-Brain, One-Seq-Liver, and Mix-Seq-Abdomen, for brain, liver, and abdominal lesion segmentation using a combination of the text and the image exemplar as prompts. As shown in the figure, incorporating previous images and their corresponding annotations as additional guidance consistently improved the final segmentation results. This observation is consistent with the quantitative results reported in Table 9, which demonstrate that temporal or sequential prior information provides useful contextual cues for more accurate lesion localization and boundary delineation.

#### Visual comparison of the segmentation results under different fine-tuning strategies

**Fig. 9:**
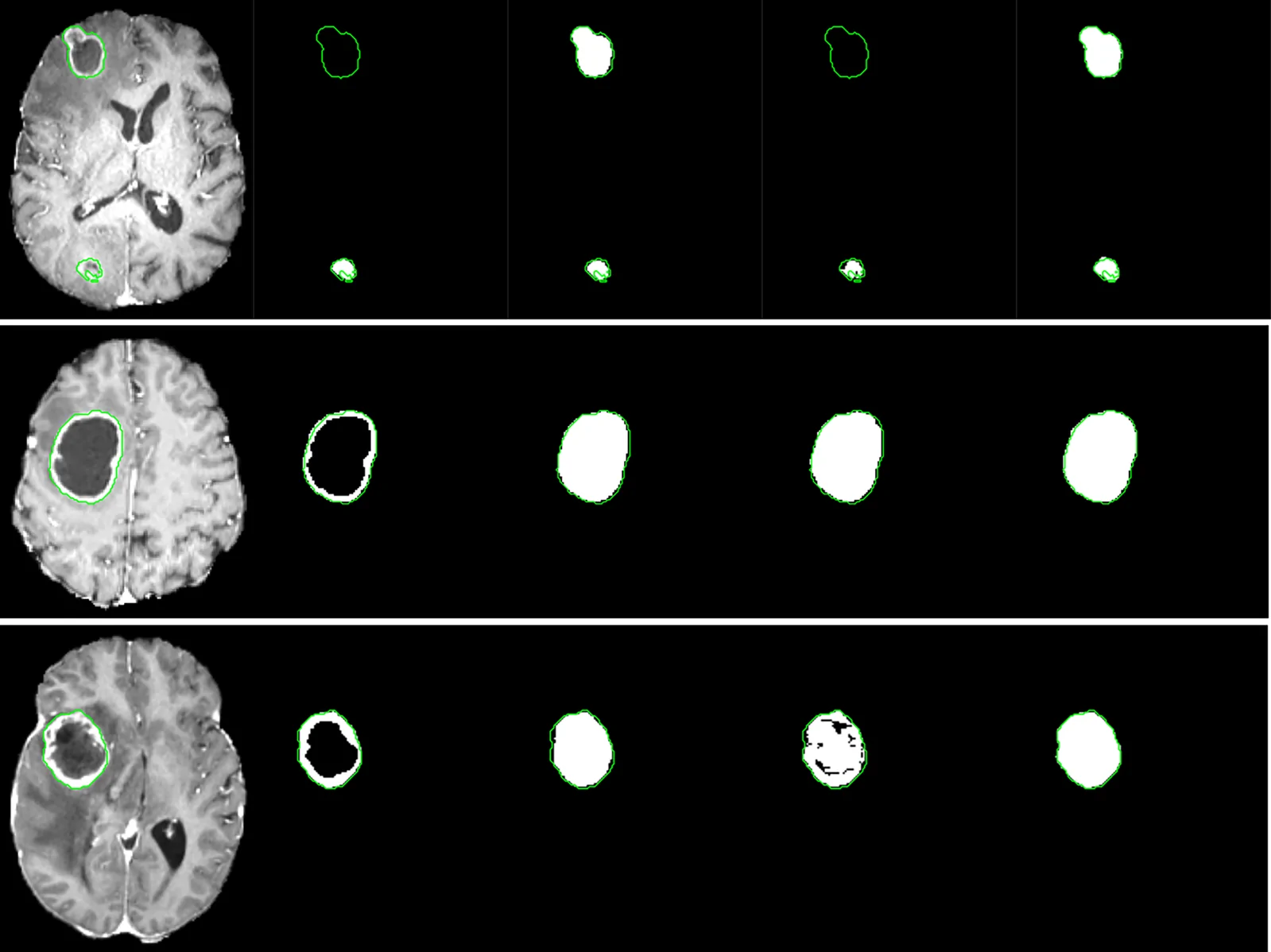
Visual segmentation results obtained with the original SAM3 (2nd column), SAM3 with full fine-tuning (3rd column), SAM3 with detector-only fine-tuning (4th column), and SAM3 with adapter-based fine-tuning (5th column). The green contours indicate the ground truth, and the white masks denote the predicted segmentations. SAM3: Segment Anything Model 3

Figure 9 presents the visual results obtained using the original SAM3 model, SAM3 with full fine-tuning, SAM3 with detector-only fine-tuning, and SAM3 with adapter-based fine-tuning on the BraTS2023-MET dataset, with combined text and image exemplar as prompts. As shown, fine-tuning consistently improves the segmentation performance over the original SAM3 model, indicating the benefit of task-specific adaptation.

Among the three fine-tuning strategies, detector-only fine-tuning yielded inferior results compared with both full fine-tuning and adapter-based fine-tuning, suggesting that updating only the detection module is insufficient for optimal lesion segmentation. Furthermore, when comparing full fine-tuning with adapter-based fine-tuning, the adapter-based approach achieved better results for this dataset. This observation suggests that with limited training data, full fine-tuning may introduce a higher risk of overfitting, whereas adapter-based fine-tuning provides a more stable and parameter-efficient alternative.

## Discussion

In this study, the lesion segmentation performance of the original SAM3 framework was evaluated using concept-based prompts. The authors also explored potential strategies to further improve the effectiveness through the integration of prior knowledge via input enhancement without additional training. Finally, multiple fine-tuning strategies for SAM3 were investigated. Although both prior knowledge augmentation and fine-tuning improve the segmentation performance of SAM3, several limitations remain and warrant investigation in future work.

### Prior knowledge integration as input

Prior knowledge such as spatial location, relative scale, and multimodal imaging information plays a critical role in accurate lesion localization and delineation. Previous studies, including SAM2-Aug [69], have demonstrated that integrating prior MR images and their corresponding annotations into SAM2 can substantially improve the tumor localization and segmentation performance in ART scenarios, where historical imaging and annotations are readily available. In this study, a similar strategy was effective for SAM3 (Table 9, Fig. 8). The effectiveness of this approach is largely attributable to limited variation in the tumor morphology and location typically between consecutive treatment sessions. However, naïvely supplementing the current input with prior MR images and their annotations may be less ideal. This strategy primarily provides coarse spatial cues and may be susceptible to additional noise, including inaccurate boundaries and deformation/motion between the current and prior imaging.

Moreover, it was found that directly using predicted masks from preceding slices of 3D volumes as a prior can improve SAM3’s segmentation performance in some, but not all, scenarios (Table 7). For example, a previous slice may contain a lesion, whereas the current slice does not, introducing misleading information and an increased risk of false-positive predictions. Similarly, using multimodal images as input generally improves segmentation performance but can also lead to decreased accuracy in some scenarios (Table 8). These observations highlight a key limitation of naïve prior-knowledge integration.

Therefore, an effective segmentation framework should be capable of distinguishing subtle discrepancies between the current image and auxiliary prior information while selectively leveraging prior knowledge to refine the segmentation results. Learning such adaptive fusion mechanisms remains important for improving the robustness, generalizability, and detection accuracy of SAM3-based lesion segmentation.

### Image exemplars prompt generation

Prompt augmentation is an effective strategy for SAM-based methods [69, 74]. This study investigated the effectiveness of lesion segmentation under concept-based prompting using text and/or image exemplars. The initial results indicate that relying solely on text prompts is insufficient for accurate lesion localization, primarily because of the low contrast of lesions and their visual similarity to the surrounding tissues or organs. Although combining text prompts with image exemplars significantly improves performance, this approach remains inefficient and potentially impractical in clinical settings because it requires the manual identification of lesion exemplars for each image slice.

To address this limitation, a simplified image exemplars prompt augmentation strategy could be explored, in which one or a small set of image exemplars from a training image or a subset of training images are reused as shared prompts across all test samples in SAM3. Specifically, in the one-shot scenario, a single-lesion image exemplar from the training set was selected and reused as the common prompt for all test samples. In a multi-exemplar scenario, a set of lesion exemplars is first constructed based on predefined selection criteria or similarity metrics. Subsequently, an automatic selection algorithm is employed to identify one or more representative exemplars from the library that serve as prompts. Compared with per-image or per-slice exemplar prompting, both strategies can substantially improve computational and annotation efficiency while still providing meaningful visual guidance to the model.

Additionally, several promising directions merit further investigation for candidate lesion detection using image exemplars as guidance. First, at the image level, anatomical priors can be leveraged to copy and place image exemplars into anatomically corresponding regions using multiscale and deformable transformations, while also accounting for intensity distribution differences between the exemplar and target images. Second, at the feature level, multilayer features extracted from the SAM3 image encoder can be exploited to compute the similarity between the exemplar and test samples, enabling automatic candidate regions or bounding-box generation for adaptive exemplar prompting. Third, it may be beneficial to design and train a dedicated prompt-generation module or to leverage the streaming memory mechanism of SAM3, allowing the model to capture temporal relationships across frames and track lesions using a single prompt.

Finally, image exemplars were randomly selected as prompts to provide a simple and unbiased baseline setting that did not rely on prior assumptions regarding exemplar quality or selection criteria. However, the development of optimized exemplar selection mechanisms to identify the most informative image exemplar for each target case is a promising direction for future research. Achieving this objective would likely require constructing an exemplar repository and designing retrieval strategies at either the image or feature levels to identify the most suitable exemplar. It is believed that appropriately selected exemplars can provide richer prior knowledge and a more relevant semantic context, thereby offering stronger guidance for lesion detection and segmentation and ultimately improving model performance.

### Automatic prompt generation

Automatic high-quality prompt generation is essential. More broadly, there are currently two major approaches for reducing human interaction in prompt-based foundation models. The first seeks to eliminate prompts entirely by replacing the original prompt encoder with learnable modules or integrating the pretrained image encoder into segmentation architectures. Representative examples include AutoSAM [75], Semantic AutoSAM [76], SAM2-UNet [77], and nnSAM [78]. These approaches effectively transform prompt-driven foundation models into task-specific segmentation networks that are capable of directly generating segmentation masks without user interaction.

The second approach aims to preserve the prompt-driven paradigm while automatically generating prompts, thereby reducing manual effort without sacrificing the flexibility of interactive segmentation. Representative examples include SAMUS [79], YOLO-SAM2 [80], and APSAM [81]. Compared to prompt-free approaches, automatic prompt generation maintains compatibility with the original foundation model design and allows human-in-the-loop refinement. However, their performance is highly dependent on the quality and robustness of the generated prompts.

### Fine-tuning for SAM3

Fine-tuning is essential to mitigate the domain gap between natural images and medical imaging data when adapting foundation models [31, 39]. The authors investigated different fine-tuning strategies for SAM3 and found that the one focused on lightweight adapter modules was the most effective. Although this approach demonstrated performance improvements on a single dataset, several limitations remain.

First, the issue of knowledge degradation after fine-tuning requires further investigation. Performance, especially on generalizability, can drop following fine-tuning, raising concerns about catastrophic forgetting, particularly when fully fine-tuning large pretrained models. Developing strategies to preserve generalizable knowledge while adapting to domain-specific tasks remains challenging. Second, in this study, adapter modules were inserted into the image encoder based on empirical design choices. However, principled guidelines for determining the components of SAM3 that should be fine-tuned are lacking. For example, it remains unclear whether fine-tuning should also involve the prompt encoder, detector, or mask decoder, and how such choices affect performance, stability, and generalization. Finally, a joint exploration of fine-tuning and model distillation may offer promising directions. Such a strategy not only enhances model generalization and segmentation accuracy but also facilitates deriving compact and efficient models suitable for deployment in resource-constrained or edge-computing environments.

Additionally, a substantial performance degradation was observed when using only a text prompt (i.e., ‘lesion’), suggesting that fine-tuning the text encoder and better aligning its representations with those of the image encoder are essential for achieving effective lesion segmentation. This finding indicates that developing large-scale vision-language foundation models specifically tailored for medical imaging is a promising research direction. However, jointly optimizing and continuously adapting text and image encoders while maintaining effective cross-modal feature alignment remains an open challenge. Designing more informative and task-specific text prompts to better exploit semantic priors represents another promising avenue for improving segmentation performance.

### Uncertainty modeling and human-based prior knowledge

Binary lesion segmentation is often an ill-defined problem. Clinically, lesions can be categorized as conspicuous lesions (CLs) or nonconspicuous lesions (nCLs) [41]. This study focused on CL, for which binary annotations are typically regarded as the default ground truth. However, in practice, lesion boundaries are rarely crisp or binary. Both CL and nCL often exhibit gradual transitions from regions with high diagnostic confidence to areas with lower certainty. Consequently, modeling the uncertainty or fuzziness of lesion boundaries is valuable, as it may provide additional information that can assist in clinical interpretation and decision-making. Several studies [41, 82, 83] have explored disease quantification without explicit object delineation to improve the efficiency of clinical workflows. In addition, inspired by recent advances in vision-language modeling, clinically relevant knowledge can be integrated into segmentation frameworks. For example, textual descriptions can capture modality-specific characteristics of lesions and encode expert knowledge regarding their appearance across different imaging modalities. Lesions that appear clearly on CT may present differently on positron emission tomography, and vice versa. Therefore, incorporating human-based prior knowledge in textual form to describe such cross-modality differences may improve segmentation accuracy and efficiency.

In addition, the prior knowledge fusion strategy could be further improved by employing more selective or adaptive mechanisms rather than the simple concatenation adopted in this study. Future work may explore more sophisticated fusion architectures, such as adaptive attention-based fusion modules or dedicated cross-modal interaction branches, as well as task-specific regularization strategies, to better integrate prior knowledge with image features. Such approaches can encourage the model to leverage high-level semantic information more effectively, leading to more efficient representation learning and improved lesion detection and segmentation performance.

## Conclusions

In this study, a comprehensive evaluation of SAM3 is presented for concept-based lesion segmentation across different medical imaging modalities and diverse anatomical regions, with particular emphasis on volumetric medical imaging datasets. Thirteen datasets encompassing 11 lesion types were investigated to assess the generalization capability, robustness, and limitations of SAM3 under text- and exemplar-based prompting to reduce the lesion-wise prompting burden. The results demonstrate that SAM3 exhibits strong cross-dataset and cross-modality generalization, although performance variability was observed across different lesion types and imaging modalities. By integrating prior knowledge and employing PEFT strategies, segmentation accuracy and robustness can be further improved. These findings suggest that concept-driven vision-language foundation models hold significant promise for scalable lesion segmentation and may serve as a foundation for more flexible and annotation-efficient clinical workflows.

## Supplementary Information

Below is the link to the electronic supplementary material.

Supplementary Material 1

## Data Availability

The datasets used and/or analyzed during the current study are available from the corresponding author on reasonable request.

## Footnotes

1 In this study, the term “lesion” is adopted to denote a broad category of abnormal tissue regions, including tumors, polyps, ischemic stroke lesions, and other pathological findings across different organs, tissue regions, and conceptual regions such as lymph node zones.

2 https://www.vecteezy.com/vector-art/19850122-anatomical-structure-human-body.

3 In the original SAM3 supplementary material, the module responsible for fusing visual and textual features is referred to as the Multimodal Decoder. In this work, the term vision-language fusion module (V-L Feature Fusion) is adopted to maintain consistency with the dual encoder-decoder nomenclature used throughout the paper and to better emphasize its architectural role and functional purpose within the dual encoder-decoder framework.

## Abbreviations

ADC: Apparent diffusion coefficient
ART: Adaptive radiation therapy
ASD: Average surface distance
BraTS: Brain tumor segmentation
BUSI: Breast ultrasound image
CL: Conspicuous lesion
CNN: Convolutional neural network
CT: Computed tomography
DWI: Diffusion-weighted imaging
HD95: 95th-Percentile Hausdorff distance
KiTS: Kidney tumor segmentation
LLM: Large language model
MRI: Magnetic resonance imaging
MSD: Medical segmentation decathlon
nCL: Nonconspicuous lesion
PE: Perception encoder
PEFT: Parameter-efficient fine-tuning
PVS: Promptable visual segmentation
SAM3: Segment Anything Model 3
T1-C MR: T1-contrast-enhanced MR
